# Is the Voronoi Entropy a True Entropy? Comments on “Entropy, Shannon’s Measure of Information and Boltzmann’s H-Theorem”, *Entropy* 2017, *19*, 48

**DOI:** 10.3390/e21030251

**Published:** 2019-03-06

**Authors:** Edward Bormashenko, Mark Frenkel, Irina Legchenkova

**Affiliations:** Engineering Faculty, Chemical Engineering, Biotechnology and Materials Department, Ariel University, P.O.B. 3, 407000 Ariel, Israel

**Keywords:** Voronoi entropy, pattern, thermodynamic entropy, Shannon measure of information, intensive value

## Abstract

The goal of this comment note is to express our considerations about the recent paper by A. Ben Naim (*Entropy* **2017**, *19*, 48). We strongly support the distinguishing between the Shannon measure of information and the thermodynamic entropy, suggested in the paper. We demonstrate that the Voronoi entropy should also be clearly distinguished from the entropy of a two-dimensional gas. Actually, the Voronoi entropy being an intensive value is the averaged Shannon measure of ordering for a given pattern.

The above paper by Ben Naim [1] criticized the identification of the Shannon measure of information (abbreviated SMI) with the thermodynamic notion of entropy. We are quoting: “The first (SMI) is defined on any probability distribution; and therefore it is a very general concept. On the other hand, entropy is defined on a very special set of distributions” [1]. Actually, the thermodynamic entropy is a special case of a SMI, corresponding to the distribution that maximizes the SMI, referred to as the equilibrium distribution [1]. Thus, SMI should be clearly distinguished from the thermodynamic entropy. In our comment we exploit the arguments suggested by Ben Naim [1,2,3,4] for distinguishing between the Voronoi entropy [5] and the averaged Shannon measure of two-dimensional ordering (abbreviated further SHMO), supplied by the Shannon-like expression, introduced by Voronoi [5]. The Voronoi entropy (known already to Kepler and Descartes [6,7]) is the useful notion, enabling the estimation of ordering for the set of points (also called seeds or nuclei) located in a plane [8,9]. 

A Voronoi tessellation or diagram of an infinite plane is a partitioning of the plane into non-overlapping convex polyhedral regions based on the distance to a specified discrete set of points. For each seed, there is a corresponding region consisting of all points closer to that seed than to any other. The Voronoi polyhedron of a point nucleus in space is the smallest polyhedron formed by the perpendicularly bisecting planes between a given nucleus and all the other nuclei [8,9]. To quantify the orderliness of the Voronoi tessellation the so-called Voronoi entropy is defined as:

(1) $$S_{vor}=-\sum_{i}P_{i}lnP_{i}$$

where *P_i_* is the fraction of polygons with *n* sides or edges for a given Voronoi diagram [5,8,9]. Equation (1) has the form similar to SMI and the entropy in statistical mechanics [1]. That is why it was called “the Voronoi entropy”. Ben Naim called the identification of SMI with the thermodynamic entropy “grievous mistake” [1]. The same is true for the identification of the Voronoi entropy *S_vor_* with the thermodynamic entropy of 2D gas. This identification is erroneous for several reasons:

(1) The Voronoi entropy may be calculated for any set of points, starting from the number *N* > 3. These points may represent the 2D gas, but this gas is not necessarily in the thermal equilibrium [10] and it obviously may be time-dependent [10].
(2) The Voronoi entropy is an intensive value. This means that the Voronoi entropy of the pattern characterized with the given and constant 2D order does not depend either on the area of the pattern nor on the number of seed points (of course, this is true, when the boundary effects are neglected). In contrast, the entropy is an extensive thermodynamic value, in other words it grows with an increase in a number of particles constituting the system [11,12].
(3) The Voronoi entropy is not the relativistic invariant value. The relativistic contraction changes the pattern and simultaneously it changes the Voronoi entropy related to the pattern. Whereas the thermodynamic entropy is the relativistic invariant [13].

Therefore, what is measured by the Voronoi entropy? Following Ben Naim [1], we suggest that actually the Voronoi entropy is the averaged Shannon measure of ordering for a given pattern (SHMO). However, this definition also needs certain care. Indeed, it is reasonable to suggest that the maximal value of the Voronoi entropy corresponds to random 2D patterns, for which $S_{vor}=1.71$ was established [14,15]. Note, that we revealed recently ordered patterns, arising from the points located on the Archimedes spiral (such as shown in Figure 1), demonstrating the Voronoi entropy, which is markedly larger that $S_{vor}=1.71$, reported for random patterns [14,15,16]. Moreover, the Voronoi entropy may grow unrestrictedly with the number of kinds of polygons appearing in the pattern. The correct statement should be formulated as follows: The Voronoi entropy quantifies the ordering for the patterns demonstrating the same number of polygons.

Following Ben Naim [1], we conclude that actually the Voronoi entropy should be clearly distinguished from the thermodynamic entropy of the 2D gas, and actually it represents the averaged Shannon measure of ordering for 2D patterns.

## Figures and Tables

**Figure 1:**
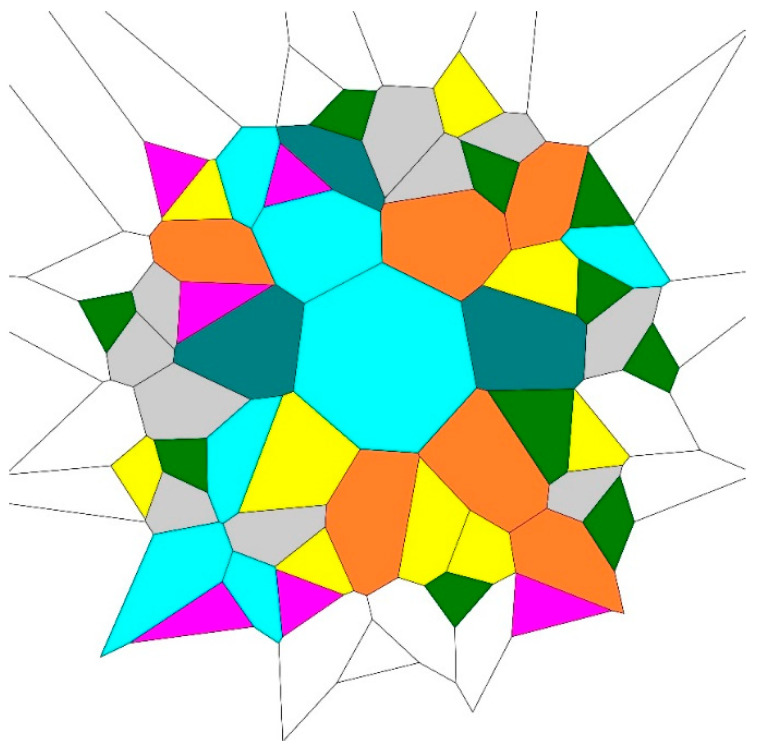
80 points pattern (the total numbers *N* = 80), built from seven types of polygons and demonstrating the Voronoi entropy *S_vor_* = 1.8878 is shown. (Color mapping: Magenta polygons are triangles, green—tetragons, yellow—pentagons; grey—hexagons, blue—heptagons; brown—octagons, deep-green—nonagons).
